# Optimization of a rotary desiccant wheel for enthalpy recovery of air-conditioning in a humid hospitality environment

**DOI:** 10.1016/j.heliyon.2022.e10796

**Published:** 2022-09-27

**Authors:** Hung-Yi Tsai, Chung-Tai Wu

**Affiliations:** Lee-Ming Institute of Technology, Taiwan

**Keywords:** Desiccant wheel, Enthalpy recovery, Moisture removal capacity (MRC)

## Abstract

Excessive condensation on cooling coils can be costly energy-wise due to humid process air streams. An innovative desiccant cooling system is introduced into an existing hotel in Taoyuan, Taiwan, where the ambient is humid year-round. Rejected heat from the existing cooling system is used as the energy source to heat the regeneration airstream for dehumidification of the process airstream via a rotary desiccant wheel. The paper develops and validates a numerical model for the heat and mass (moisture) transfer of the rotary desiccant wheel. The validated numerical model is used to simulate the expected performance of the rotary wheel under various conditions and operating strategies. The desiccant wheel’s performance includes the steady-state moisture removal capacity (MRC) and the transient response time to reach steady-state. The examined factors include the ambient temperature and humidity, air flow rate, rotational speed of the wheel, wheel-split, and regeneration airstream temperature. From the simulation results, the paper offers the optimized control strategies for the operation of the rotary desiccant wheel system in the hotel.

## 1 Introduction

With global warming and rising cost of fossil fuels, engineers have continuously explored various options of energy saving strategies for heating, ventilating, and air-conditioning (HVAC) operations. While each novel strategy presents opportunities for energy savings, each novel configuration of system components only works well under a specific niche market (e.g. hospital, food storage, underground parking) and/or an ambient environment to avoid compromised comfort, air quality, prolonged payback time, and similar others. For example, an evaporative cooling system only works well in a dry environment and the ambient temperature is not too high. A ground-coupled system would require high upfront costs of installing heat-exchangers below deep-earth (Hackel and Pertzborn, 2011). The energy saving potential of a liquid pressure amplification approach would be limited when the ambient temperature falls to a certain temperature (Al-Rabghi and Akyurt, 2004). A radiant chilled ceiling system can significantly reduce energy consumption but cannot moderate indoor humidity. A developed panel (Fauchoux et al., 2010) can overcome this limitation but gathered water condensation on the chilled panel may cause water dropping on the ceiling. An ejector cooling system usually requires a heat source of more than 80 °C and has lower coefficient-of-performance (COP) compared with conventional vapor compression systems (Cardemil and Colle, 2012). A thermal storage system only shifts energy usage from on-peak to off-peak periods to avoid peak demand charges but not energy reduction (Wu and Tsai, 2015). Although expensive in both capital and running cost, desiccant cooling systems are gradually attaining wider market penetration in a hot and humid climate.

Issues of humidity control have always been the major area of concern by HVAC engineers over the last several decades. Especially in south-east Asia where the humidity level is high year-round, high enthalpy of the moist air makes the total energy cost of removing ambient moisture a significant issue. Many vapor compression (VC) cooling units simply cannot remove sufficient amount of moisture without overcooling the building space or tagging additional costs to reheat. Moreover, damages to ventilation equipment and furnishings due to moist exposure can also be costly and time-consuming. When used in conjunction with conventional air-conditioning systems, condenser-rejected heat can be used as a free heat source to deep-dry (usually 80–120 °C) moisture content of a rotary desiccant wheel for enthalpy recovery and better humidity control of the supply air to the ventilating space.

A desiccant cooling system by ways of an adsorptive solid (Rambhad et al., 2016; Wu et al., 2018) or absorptive liquid material (Jain et al., 2011; Everly et al., 2015) is an interesting alternative to conventional vapor compression cooling units (Wang et al., 2013; Qi et al., 2013). What makes a desiccant cooling system so attractive is the minimal renewable energy source required from either thermal waste (Angrisani et al., 2015; Zendehboudi et al., 2018) or solar thermal energy (Al-Alili et al., 2014) that is typically integrated with an auxiliary fossil-fuelled system. An electric chiller would typically be used to balance the required sensible load in a hybrid desiccant cooling system (Al-Alili et al., 2014; Jani et al., 2016).

Obstacles of desiccant cooling systems may be the perceived high investment costs, low familiarity, and the lack of knowledge about the system performance and rate-of-return. Regarding system performance, the concern of relatively long response time for moisture transfer to reach steady state on the rotary wheel should definitely be addressed for optimal control. It is noted that there may be other maintenance concerns pertaining to desiccant cooling systems such as corrosive problems and/or crystallization on the silica gel (WSG) and molecular sieve (LT3) desiccant wheels but these issues can be overcome easily with adequate care. Nonetheless, an optimized desiccant cooling approach has the potential for improved energy efficiency and reduced environmental impact. Extra upfront costs can be rewarded with substantial savings in the long haul under a desiccant optimization strategy.

Plenty of desiccant cooling studies exist as mentioned above. However, researches that predict and characterize transient response of rotary desiccant wheels for dehumidification have been limited. Although some have used an average NTU for an entire stream or wedge, the difference in NTU between inlet states for the process and regeneration streams can be substantial. The study attempts to use finite difference method at every element, recognizing the set of finite difference equations that fit the tri-diagonal matrix patterns. Solution of the tri-diagonal matrix is much faster and more efficient than simple matrix inversion techniques. This can then be solved iteratively until convergence is achieved. Hopefully, the developed model may predict the transient response with improvements to previous efforts. In all, the study may fill the gap of previous research efforts by answering two specific research questions, outlined as:

RQ1 How accurately does a set of finite difference equations fit the tri-diagonal matrix patterns to portray the transient nature of rotary desiccant wheels? (Numerical aspect)

RQ2 How well does a rotary desiccant wheel actually work, performance-wise, in a hot and humid climate? (Experimental aspect)

To answer these research questions, the study reviews the utilization of an installed rotary desiccant wheel cooling system in a hotel of hot and humid climate through experimental and analytical analysis. In order to predict and characterize the transient response of the desiccant wheel, a computer model is developed. Experimental tests are performed to validate the numerical model, at both the process airstream outlet and the regeneration airstream outlet. After validation, a parametric analysis is followed to characterize the transient response of the desiccant wheel at various scenarios (e.g. different ambient conditions, wheel speeds, etc.). At last, control strategies are explored for more efficiently-operated transient response of the desiccant cooling system, as well as the optimization of the steady-state performance.

## 2 Literature review

In the pursuit of reducing moisture content of an occupied space while maintaining energy performance, researchers have long advocated decoupling dehumidification and cooling to make humidity control independent from temperature control (Jain et al., 2011; Zhang and Lee, 2011). A popular approach that uses desiccant dehumidification with chilled ceiling and displacement ventilation could save 8.2% of total primary energy consumption when compared with conventional all-air systems (Hao et al., 2007). A hybrid system using chilled beams, absorption refrigeration, and desiccant dehumidification powered by solar energy could save up to 36.5% of annual primary energy consumption (Fong et al., 2011). A proposed hybrid chilled-ceiling system with desiccant cooling could theoretically save up to 44% of primary energy consumption but high possibility of condensation on the chilled ceiling surface exists in hot and humid climates (Niu et al., 2002). Another problem associated with chilled-ceiling systems is that, with radiant cooling, less dense cool air would naturally drop down from the ceiling to suppress the stratification boundary of displacement ventilation to the occupied space, causing thermal discomfort (Novoselac and Srebric, 2002). Furthermore, the source of chilled water comes from vapor compression refrigeration via a mixing arrangement to raise the desired secondary loop temperature which wastes energy in chilled ceiling systems. Hence, chilled ceiling technique is more viable in a cooler climate such as in northern Europe (Costelloe and Finn, 2003).

The key to desiccant wheel cooling is the source of thermal energy required to dehumidify moist air, be it an electric heat pump (Hwang et al., 2017), a solar collector (Everly et al., 2015), or heat recovery from gas-fired engines (Angrisani et al., 2015). Solar radiation is an attractive heat source that is both free and clean, but the viability of a solar desiccant cooling system is highly dependent of the site-specific conditions and the loading rate. Although solar desiccant cooling systems have been proven feasible theoretically in climates of high solar radiation (>15 MJ/m^2^ per day) (Rachman et al., 2014), payback analysis need to be addressed.

Desiccant wheel is the heart of energy recovery in desiccant cooling where silica gel coating is the most widely used material for adsorption of moist air. Although adsorbent characteristics greatly impact the performance of solid desiccant wheels, the adsorbent performance need to be appraised in a holistic approach (Fong and Lee, 2018; Wu et al., 2018). Under the conditions of heat and mass transfer analogy, specific wheel characteristics and the number-of-transfer-unit (NTU) varied entropy generation number have been studied extensively (Giannetti et al., 2015). The effect of air temperature, velocity, wall thickness, and rotary speed were clarified, with only 3.3% and 10.8% error between predicted and measured distribution for humidity and temperature, respectively (Yamaguchi and Saito, 2013). Niu and Zhang (2002) concluded that air dehumidification is more sensitive to rotary speed than enthalpy recovery, increased wheel thickness leads to lower performance, and only the outer active layer has an effect on the processed air under optimal rotational speed. The percentage of dehumidification is greater for an arrangement of counter flow than parallel flow (Narayanan et al., 2011).

In HVAC systems, the performance of cooling equipment is commonly characterized by the parameter of COP which is the ratio of cooling capacity to energy input. A conventional refrigeration cycle considers only the chiller (cooling coils) or electric COP for electric energy input. A desiccant cooling system may take into account of the thermal COP (heat input for regeneration), the sorptive COP (not covered by the cooling coils) or the overall COP (inclusive of the cooling coils). A review of past studies pertaining to COP of desiccant cooling is presented as follows.

Using the recovered heat from a Combined Heat and Power (CHP) system, the sorptive COP varied in the range of 0.41–1.34 for a high efficient combination of vapor compression vapor and desiccant wheel (Henning et al., 2007). The electricity saving of the CHP system would vary in the range of 25.6–34.3% with respect to a reference system. Under the minimum investigated regeneration temperature (60 °C), the maximum thermal COP could be in the range of 0.25–0.29. In a separate study of two operating cycles using either outdoor air or building return air for regeneration, the thermal COP increased to about 0.34 when either outdoor temperature or humidity increased (Heidarinejad and Pasdarshahri, 2011).

In the design parameters of Panaras et al. (2011), regeneration temperature, operation cycle, airflow rate, and subsystem level of performance, the thermal COP would decrease with increased regeneration temperature where the thermal COP was higher for ventilation cycle (max COP = 0.5) than the re-circulation (max COP = 0.4). The ventilation cycle COP can be as high as 0.85 for high level performance of the desiccant motor. Previous experimental findings were consistent that thermal COP increases with decreased regeneration temperature, increased air flow rate, and increased rotational speed of the desiccant wheel (Heidarinejad and Pasdarshahri, 2010). The theoretical framework of Nobrega and Brum (2011) found thermal COP to reach as high as 0.4, with a regeneration temperature of 110 °C and effectiveness of the wheel at 0.9. Experimental investigation of a designed desiccant cooling system found its COP to vary from 0.4 to 4.0 during the day, with a mean value of 1.35 (Hurdogan et al., 2010). Compared with a vapor compression system, experimental findings for various climates found the overall desiccant cooling COP to decrease about 36% in a hot-dry climate and 28% in a hot-humid climate (Mandegari and Pahlavanzadeh, 2010).

## 3 Desiccant facility

**Figure 1:**
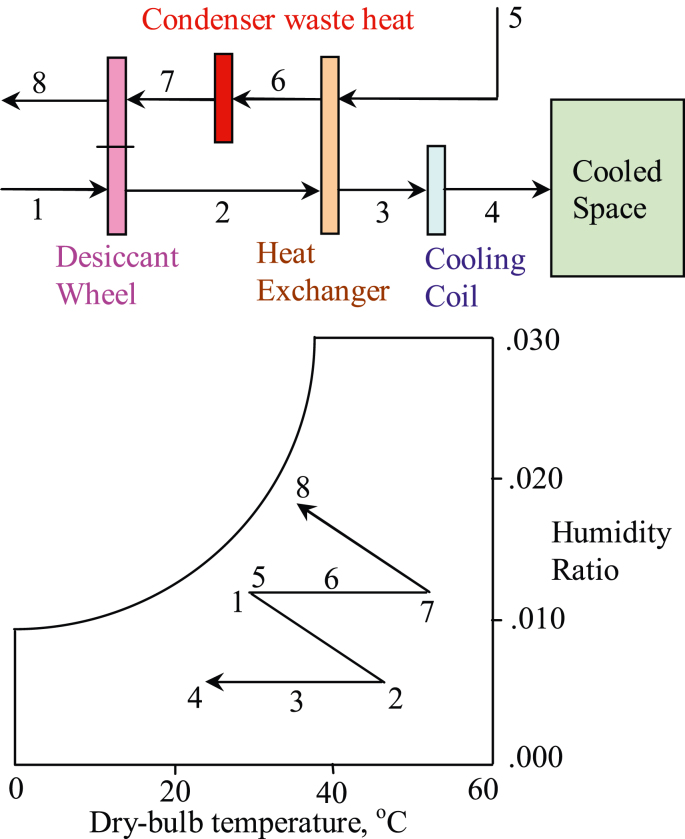
Psychrometric process of the desiccant enhanced cooling cycle.

In many desiccant cooling systems, after fresh supply air is deep-fried (dehumidified) by a rotary desiccant wheel, an enthalpy recovery wheel that rotates between the process air (dehumidified hotter air) and return air (cooler air) would cool down the processed supply air (Al-Alili et al., 2014). However, an enthalpy wheel is not suitable for the hospitality operation in Taoyuan Hotel (situated 15 km south of the Taoyuan International Airport in Taiwan) where high latent heat persists throughout the building, as well as its ambient (subtropical) climate. A commercially available desiccant wheel (diameter = 1.22 m, depth = 0.2 m) by Novel Aire Technologies was inserted within the air-handling-unit (AHU) as parts of the hotel’s major renovation in 2016. Heat rejection from super-heated refrigerant is used as the heat source to dehumidify moist air via a heat exchanger before going through the cooling tower. A schematic drawing of the hotel’s desiccant cooling cycle and the corresponding state of the air temperature and humidity on the psychrometric chart are shown in Figure 1, where state “1” is the process air at the inlet of the rotary wheel, state “2” is the process air at the outlet of the rotary wheel (i.e. the dehumidified supply air to be cooled), state “7 is the regeneration air at the inlet of the rotary wheel, and state “8” is the regeneration air at the outlet of the rotary wheel (i.e. the exhaust air). Adiabatic process from state “7” to state “8” works like evaporative cooling on the regeneration side of the rotary wheel (moisture adsorbed from the process airstream is rotated to the regeneration side of the wheel and evaporated) while the process air side works like adiabatic heating (or dehumidification) going from state “1” to state “2”.

The wheel operates with 50% of the area for process airstream and the other 50% for regeneration airstream, with a total effective cross-sectional area at 0.066 m^3^. It is noted that under special circumstances, a 75/25 wheel-split (process vs. regeneration) may be used. Because the primary goal of the desiccant cooling system is the removal of the largest quantity of moisture with the most efficient use of heat input, silica gel (WSG) was chosen for the desiccant wheel instead of molecular sieve (LT3) due to the fact that the isotherm curve for WSG desiccant is more linear and rises to a higher capacity at higher humidity, whereas the isotherm curve by LT3 exhibits higher desiccant capacities at lower humidity.

## 4 Methodology

**Figure 2:**
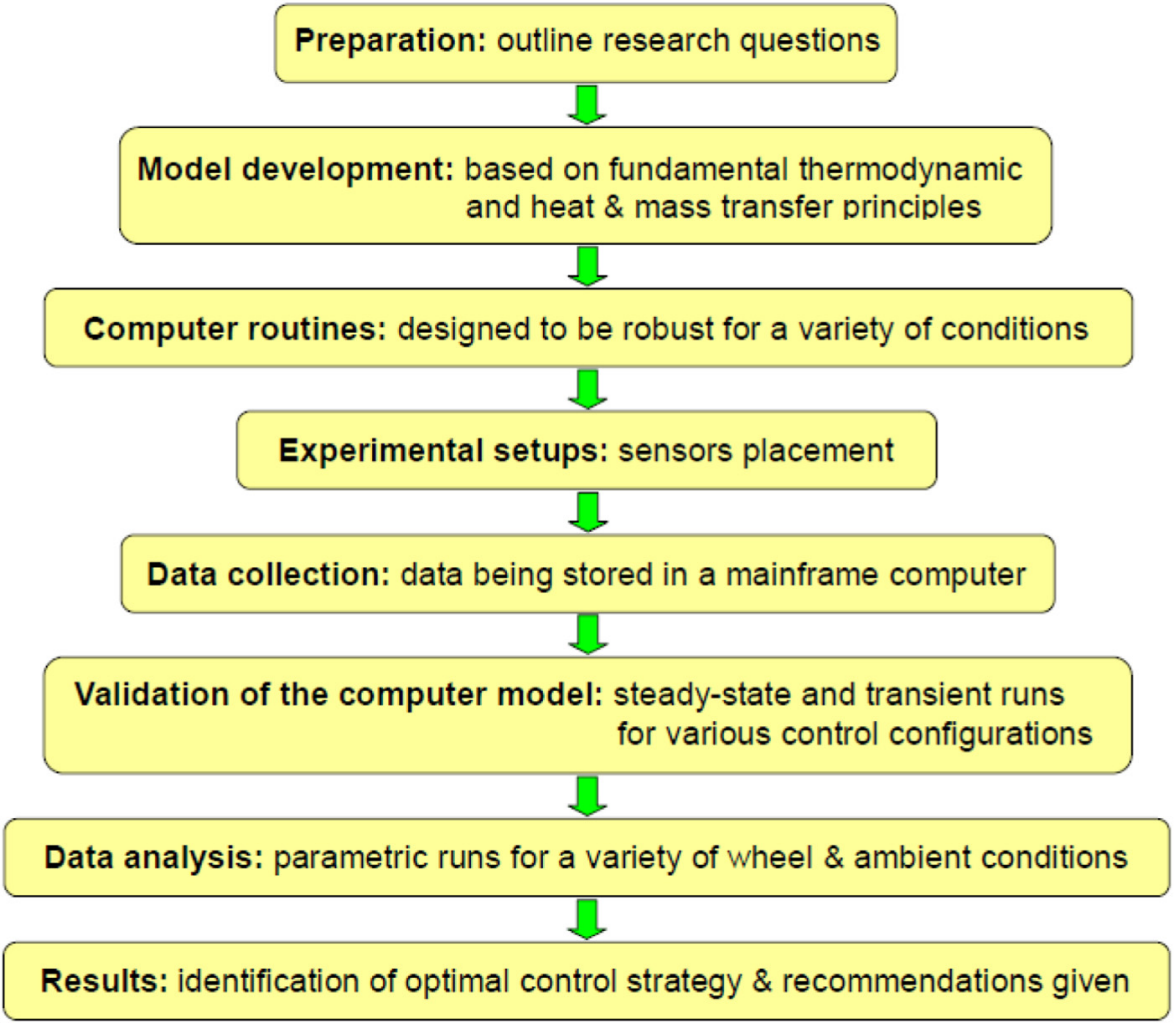
Methodological procedure.

To answer specific research questions proposed earlier, the study would follow a simplified methodological procedure shown in Figure 2. Most significant steps of the flow chart are the development of mathematical model and the validation of the computer model. For model development, the governing assumptions include: (1) the moist air behaves as an ideal gas; (2) there is a layer of moist air at the surface of the desiccant that exists in equilibrium with the desiccant bed; (3) the thermal and mass transport resistance of the matrix material is infinite in both the tangential and axial direction (minimal in the radial direction); (4) the thermal properties of the matrix material are constant; (5) temperature gradients within the desiccant particles are negligible; (6) the thermal and mass storage capacities of the air in the desiccant process are negligible in comparison with the convective heat and mass transport; (7) there is no leakage, carryover, or mixing of the two counter-flowing airstreams (process and regeneration); (8) the hysteresis effect for sorption and de-sorption is negligible. For validation of the computer model, rigorous experimental runs would be compared with simulated numbers repeatedly. *In situ* real-time dataset would be compared with numerically-generated results^1^, for both steady-state and transient response analyses due to step-changes of regeneration temperature, wheel speed, and airflow rate. After validation of the computer model, parametric analysis would be performed to identify the most optimal control strategy for the rotary desiccant wheel, with respect to ambient conditions, wheel geometrics, wheel material properties, and operational factors.

## 5 Mathematical model

The governing equations of heat and mass transfer for a rotary desiccant wheel may consider the rotating wheel as a stationary body moving through two separate air streams and boundary conditions. Presuming the desiccant wheel’s temperature (*T*) is higher than the airstream temperature (*t*) during dehumidification process, the airstream humidity (*w*) is also most-likely higher than its future equilibrium humidity (*w*_*eq*_). Hence, the mass and energy transfer rate equations (Eqs. (1) and (3)) define the differential between the airstream (*t*, *w*) and the equilibrium state of the desiccant wheel (*T*, *w*_*eq*_). However, the mass and energy conservation equations (Eqs. (2) and (4)) define the initial properties of the airstream matrix (*w*, *h*, with respect to *x*) and the wheel matrix (*W*, *hs*, with respect to *θ*).

(1) $$\text{Mass ​transfer ​rate: ​}\frac{\partial w}{\partial x}=NTU_{m,j,k}\cdot \left(w-w_{eq}\right)$$

(2) $$\text{Mass ​conservation: ​}\frac{\partial w}{\partial x}+\beta_{as}\cdot \Gamma_{as}\cdot \frac{\partial W}{\partial \theta }=0$$

(3) $$\text{Mass ​conservation: ​}\frac{\partial h}{\partial x}=NTU_{q,j,k,c_{p,ma}}\cdot \left(T-t\right)+h_{fg}\cdot \frac{\partial w}{\partial x}$$

(4) $$\text{Energy ​conservation: ​}\frac{\partial h}{\partial x}+\beta_{as}\cdot \Gamma_{as}\cdot \frac{\partial hs}{\partial \theta }=0$$

Moist air as an ideal gas is a mixture of dry air and water, where the enthalpy of moist air is the sum of the dry air enthalpy and the mixture water vapor. In terms of dry air and humidity ratio, the moist air enthalpy may be expressed as follows (Kreider and Rabl, 1994, page 124):

(5) $$h=h_{da}+w\cdot h_{wv}=c_{p,da}\cdot t_{da}+w\cdot \left(h_{fg}+c_{p,wv}\cdot t_{da}\right)$$

By defining the moist air’s specific heat as (ASHRAE, 1993, page 5.11):

(6) $$c_{p,ma}=c_{p,da}+c_{p.wv}\cdot w$$

Then, the rearrangement of Eq. (5) becomes:

(7) $$h=c_{p,da}\cdot t_{da}+h_{fg}\cdot w$$

Expressing Eq. (7) in a matrix form:

(8) $$\frac{\partial h}{\partial x}=c_{p,da}\cdot \frac{\partial t_{da}}{\partial x}+h_{fg}\cdot \frac{\partial w}{\partial x}$$

Inserting Eq. (8) into the energy transfer rate equation (Eq. (3)), the enthalpy of vaporization term and the specific heat of moist air would cancel out, hence leaving the energy transfer differential equation to be:

(9) $$\frac{\partial h}{\partial x}=NTU_{q,as}\cdot \left(T-t\right)$$

Through similar derivation, the enthalpy of the desiccant wheel expressed by a function of the desiccant matrix, water absorbed, and the integral heat of wetting (difference between the heat released by sorption and the vaporization of pure water) can be defined as (ASHRAE, 1993, page 5.11):

(10) $$hs=\left(c_{p,dm}+c_{p,lw}\cdot W\right)\cdot T+\left(h_{fg}-h_{ad}\right)\cdot W$$

Taking the derivative of Eq. (10) with respect to the wheel matrix (i.e. normalized time), Eq. (10) becomes:

(11) $$\frac{\partial hs}{\partial \theta }=c_{p,m}\cdot \frac{\partial T}{\partial \theta }+\left(h_{fg}-h_{ad}\right)\cdot \frac{\partial W}{\partial \theta }$$

Incorporating the mass conservation equation (Eq. (2)), the moist air enthalpy equation with respect to the axial distance (Eq. (8)), and the desiccant wheel surface enthalpy equation (Eq. (11)) into the energy conservation equation (Eq. (4)), canceling the enthalpy of water vaporization and moving the specific heat of the matrix to the right-hand side, the temperature matrix of the desiccant wheel can be defined as:

(12) $$\frac{\partial T}{\partial \theta }=\frac{-\left(c_{p,ma}\cdot \frac{\partial t}{\partial x}+h_{ad}\frac{\partial w}{\partial x}\right)\cdot hs}{c_{p,m}\cdot \beta_{as}\cdot \Gamma_{as}}$$

In summary, the four basic differential equations characterizing the transfer of temperature and water mass gradient of the desiccant wheel are: Eq. (1), Eq. (2), Eq. (9), and Eq. (12).

**Figure 3:**
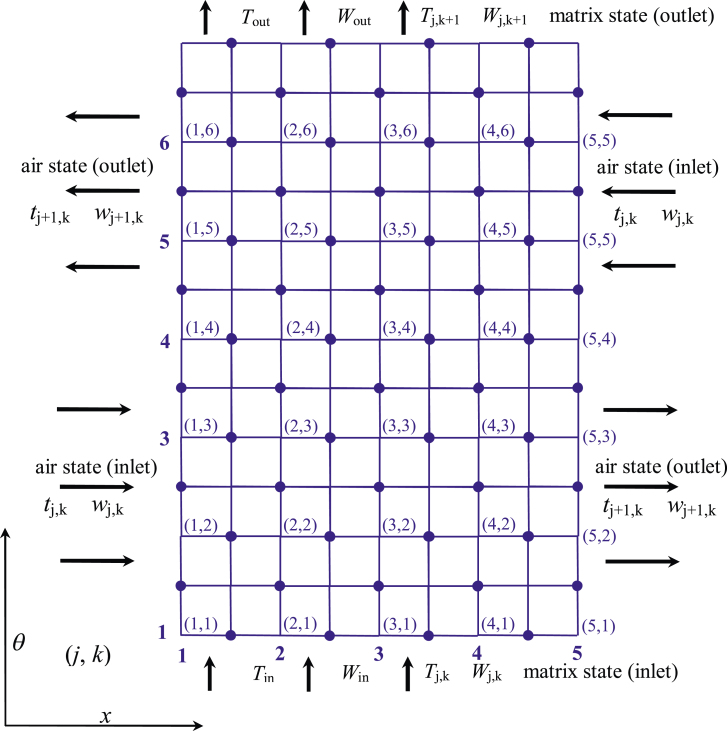
Schematic representation of a two-dimensional grid on the rotary wheel.

The finite difference mathematical model of the rotary desiccant wheel would be based on a two-dimensional grid, as shown in Figure 3. Presuming the wheel as a body rotating through time, the abscissa (*x*) would be the axial dimension while the ordinate (*θ*) would be the angular position or time. A central differencing scheme (Holmberg, 1979) would be adapted instead of forward or backward differencing techniques. From Grumbach (1999, pp. 60–62), the finite central-difference equations of the rotary wheel may be as follows:

Mass transfer rate:

(13) $$w\left(j+1,k\right)-w\left(j,k\right)=\frac{\Delta x}{2}\cdot NTU_{m,j}\cdot \left[w\left(j+1,k\right)+w\left(j,k\right)-w_{eq}\left(j,k+1\right)-w_{eq}\left(j,k\right)\right]$$

Conservation of mass:

(14) $$W\left(j,k+1\right)-W\left(j,k\right)=\frac{\left[w\left(j,k\right)-w\left(j+1,k\right)\right]\cdot \Delta \theta }{\beta_{as,j}\cdot \Gamma_{as,j}\cdot \Delta x}$$

Energy transfer rate:

(15) $$h\left(j+1,k\right)-h\left(j,k\right)=\frac{\Delta x}{2}\cdot NTU_{q,j}\cdot \left[T\left(j,k+1\right)+T\left(j,k\right)-t\left(j+1,k\right)-t\left(j,k\right)\right]$$

Conservation of Energy:

(16) $$T\left(j,k+1\right)-T\left(j,k\right)=\frac{\left[t\left(j,k\right)-t\left(j+1,k\right)+\frac{h_{ad}}{c_{p,m}}\cdot w\left(j,k\right)-\frac{h_{ad}}{c_{p,m}}\cdot w\left(j+1,k\right)\right]\cdot \Delta \theta }{\beta_{as,j}\cdot \Gamma_{as,j}\cdot \Delta x}$$

The parameters of concern are the specific properties transferred between the processed and regeneration air streams (i.e. moisture transfer by temperature differential, humidity ratio differential, and enthalpy differential). In terms of temperature differential, humidity differential, and enthalpy differential, the rotary wheel effectiveness may be defined as follows (Grumbach, 1999, page 63):

(17) $$\text{By ​temperature ​differential: ​}\varepsilon \left(t\right)=\frac{{\dot{m}}_{as,j}\cdot \left(t_{\text{process ​air, ​in}}-t_{\text{process ​air, ​out}}\right)}{{\dot{m}}_{as,\min}\cdot \left(t_{\text{ ​process ​air, ​in}}-t_{\text{regeneratiom ​air, ​in}}\right)}$$

(18) $$\text{By ​temperature ​differential: ​}\varepsilon \left(w\right)=\frac{w_{\text{process ​ ​air, ​out}}}{w_{\text{process ​ ​air, ​in}}}$$

(19) $$\text{By ​enthalpy ​differential: ​}\varepsilon \left(h\right)=\frac{{\dot{m}}_{\text{process ​ ​air}}\cdot \left(h_{\text{process ​air, ​in}}-h_{\text{process ​air, ​out}}\right)}{{\dot{m}}_{as,\min}\cdot \left(h_{\text{ ​process ​air, ​in}}-h_{\text{regeneratiom ​air, ​in}}\right)}$$

It is noted that the denominators of Eqs. (17) and (19) are not the difference of the inlet conditions. Because the process and regeneration airstreams usually come from the same source (i.e., the ambient air) that would have made the difference of humidity ratios being zero in the denominator and the subsequent effectiveness being infinite, for Eqs. (17) and (19), the maximum possible transfer of moist air in the denominator is designated as the moisture flow rate of either airstream.

## 6 Validation

The purpose of developing a validated numerical model for optimal operation of a rotary desiccant wheel is that a validated model saves both time and money over repeated experimental runs for producing predicted results. Hence, this Section is dedicated to illustrate the validity of the numerical model under both steady-state and transient modes of the operating conditions. Table 1 lists six experimental runs with comparisons of their respective numerical runs under steady-state conditions when the rotary wheel speed was set to 18 revolutions per hour (rph) while the process and regeneration air flow rates were set to 0.470 kg/s and 0.155 kg/s, respectively. It can be seen from Table 1 that the predicted numerical values of process and regeneration air temperature (*t*) and humidity ratio (*w*) are relatively close to the experimental values in that the percentage differences are within 0.94–1.79%. Generally speaking, greater discrepancy may exist among values of the process or regeneration air humidity ratio. Due to the fact that the experimental runs do not conserve both energy and mass perfectly, it is not expected that the numerical data would match the measured data perfectly. The numerical runs are based on an ideal case scenario with no loss of energy and mass. Additionally, deviation of the energy and mass balances of the measured data can also be caused by incorrect sensor readings, be it stratification of the airstreams, leakage of one airstream to the other, or just simply the inaccuracy associated with the sensors themselves.

**Table 1:** Validation results of steady-state operating condition.

Run	Experimental Runs				Numerical Runs				Comparison of percentage difference			
	Process Air		Regeneration Air		Process Air		Regeneration Air		Process Air		Regeneration Air	
	*t* [^o^C]	*w* [kg/kg]	*t* [^o^C]	*w* [kg/kg]	*t* [^o^C]	*w* [kg/kg]	*t* [^o^C]	*w* [kg/kg]	*T* [%]	*w* [%]	*t* [%]	*w* [%]
1	60.00	0.01229	47.60	0.03500	59.32	0.01209	48.12	0.03542	1.13	1.63	1.08	1.19
2	60.85	0.01226	47.90	0.03500	59.85	0.01203	48.55	0.03548	1.64	1.88	1.34	1.35
3	60.87	0.01221	47.95	0.03495	59.89	0.01201	48.47	0.03545	1.61	1.64	1.07	1.41
4	60.14	0.01232	57.70	0.03512	59.41	0.01210	57.16	0.03576	1.21	1.79	0.94	1.79
5	62.95	0.01179	49.77	0.03559	61.84	0.01198	50.61	0.03614	1.76	1.61	1.66	1.52
6	63.10	0.01179	49.45	0.03558	62.18	0.01195	50.03	0.03608	1.46	1.36	1.16	1.39

As for the validation of transient results, the statistical test would use root-mean-square-error (RMSE) to interpret the average error between two curves over the range of interest, as shown in Eq. (20). The selected range would focus on the transient response while minimizing steady-state impact. The study adapts conventional engineering practice that steady-state is reached at 95% of the average steady-state value (equivalent of 3 time-constants). Rigorous practices use 4 time-constants (98% of steady-state value) or 5 time-constants (99% of steady-state value). The RMSE statistics (between experimental and numerical results) for transient responses (of temperature and humidity) of the process air at the outlet from step-changes (increase and decrease) of the inlet regeneration air temperature, rotary wheel speed, and process air flow rate would be summarized in a Table by the end of this section. For the time-being, examples of transient effect due to step-increase of the regeneration air temperature at the inlet, rotary wheel speed, and process air flow rate are to be illustrated in the following Figures. Due to space constraint in the paper, examples of transient effect from step-decrease of the inlet regeneration air temperature, the rotary wheel speed, and the process air flow rate would not be shown. Nonetheless, the transient effects due to decreases to the aforementioned parameters are similar to their counterparts of the step-increases. Also omitted from the paper are graphic illustrations of the transient response of the regeneration air steams at the wheel outlet from step-increase and step-decrease of the inlet regeneration air temperature, rotary wheel speed, and process air flow rate. It should be reminded that the outlet of the process airstream and the outlet of the regeneration airstream are on opposite sides of the rotary wheel, as shown in Figure 1 where location [2] is the process air outlet while location [8] is the regeneration air outlet.

(20) $$\text{RMSE}=\sqrt{\frac{\sum {\left(Y_{\text{experimential,}n}-Y_{\text{numerical,}n}\right)}^{2}}{N}}$$

**Figure 4:**
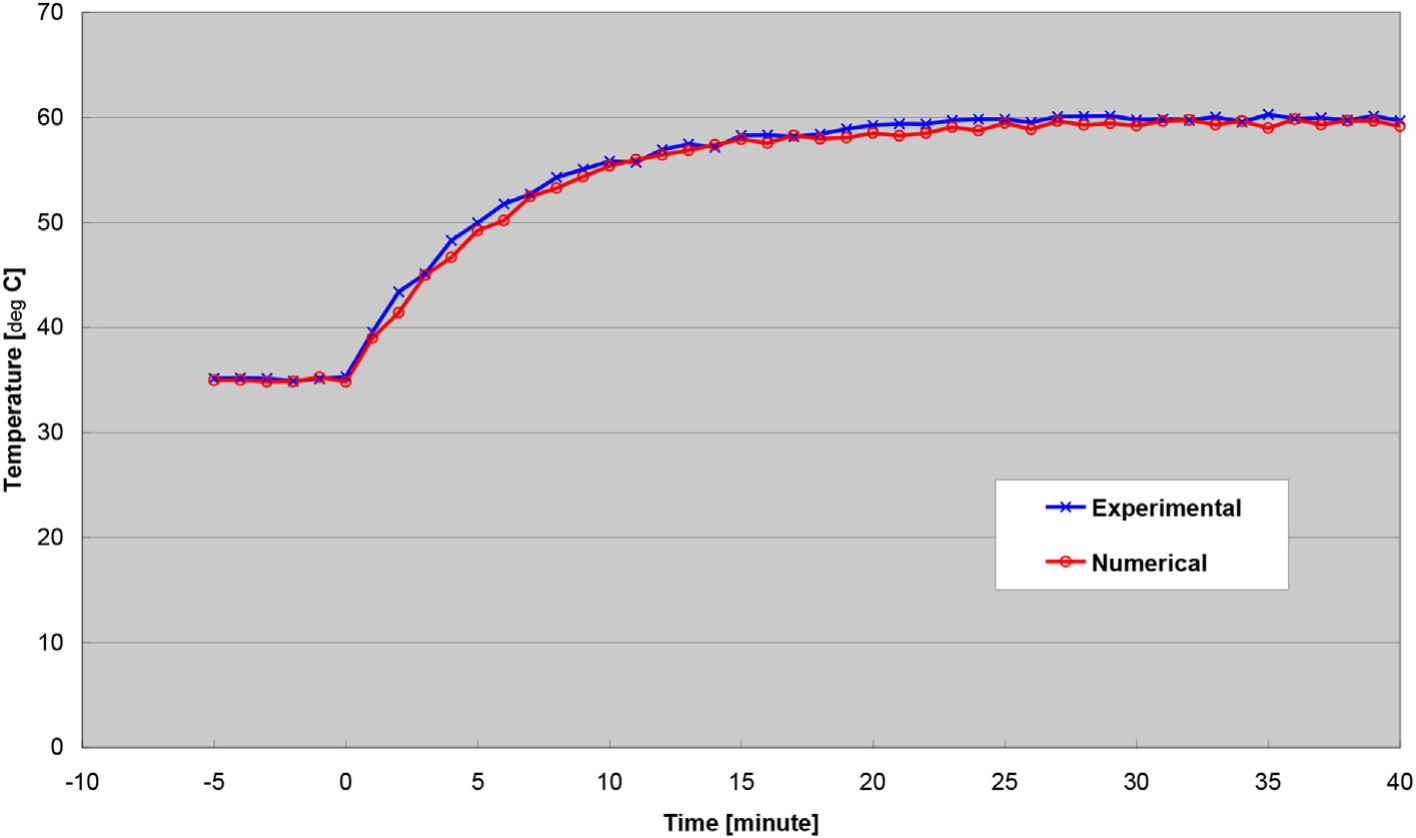
Outlet process air temperatures from increased inlet regeneration air temperature.

**Figure 5:**
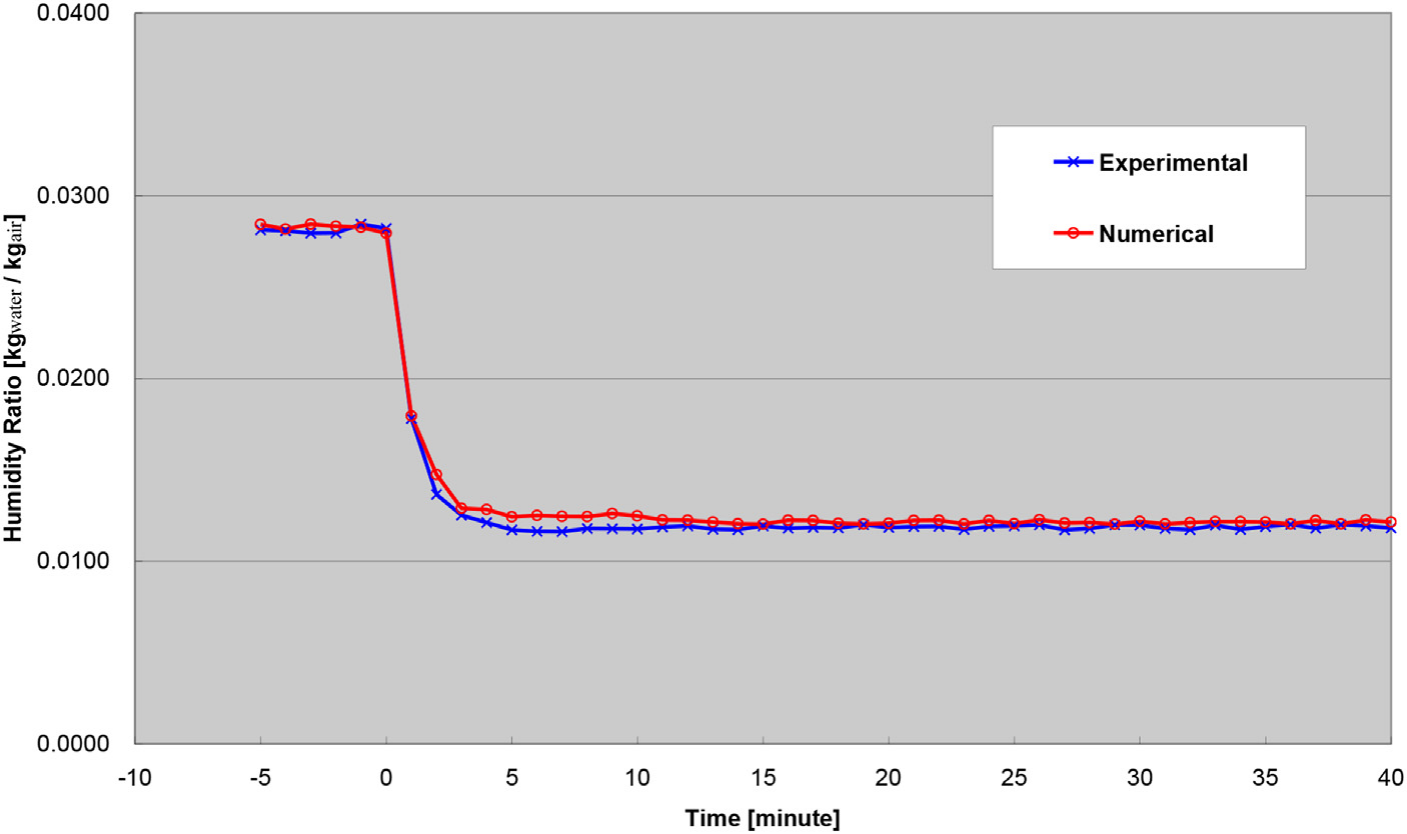
Outlet process air humidity ratios from increased inlet regeneration air temperature.

An example of the transient response at the outlet process air temperature due to an increase of the regeneration air temperature is shown in Figure 4. Likewise, from an increase of the regeneration air temperature, an example of the humidity ratio at the outlet process air as a function of time is shown in Figure 5. As seen from these Figures, the experimental and numerical curves appear to be a close match. It is noted that the discrepancy may be a little higher initially but eventually smoothes out over time for both the temperature and humidity ratio in these Figures.

**Figure 6:**
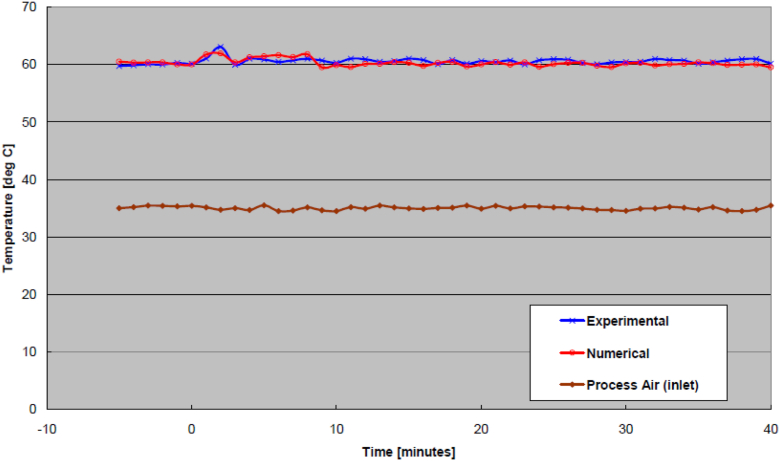
Outlet process air temperatures from increased wheel speed.

**Figure 7:**
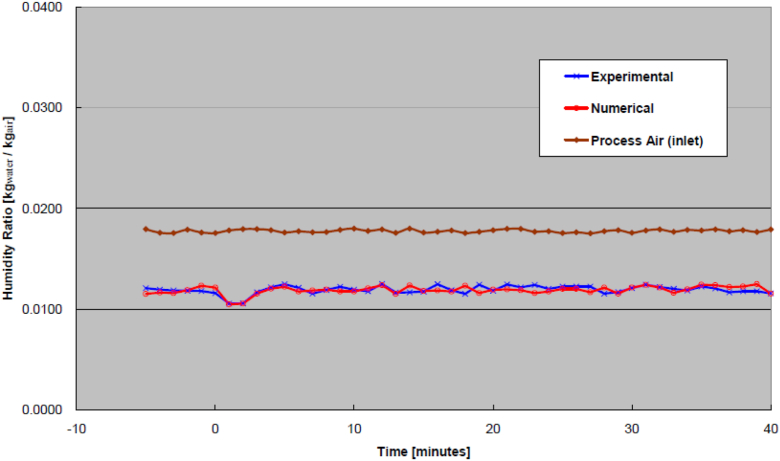
Outlet process air humidity ratios from increased wheel speed.

**Figure 8:**
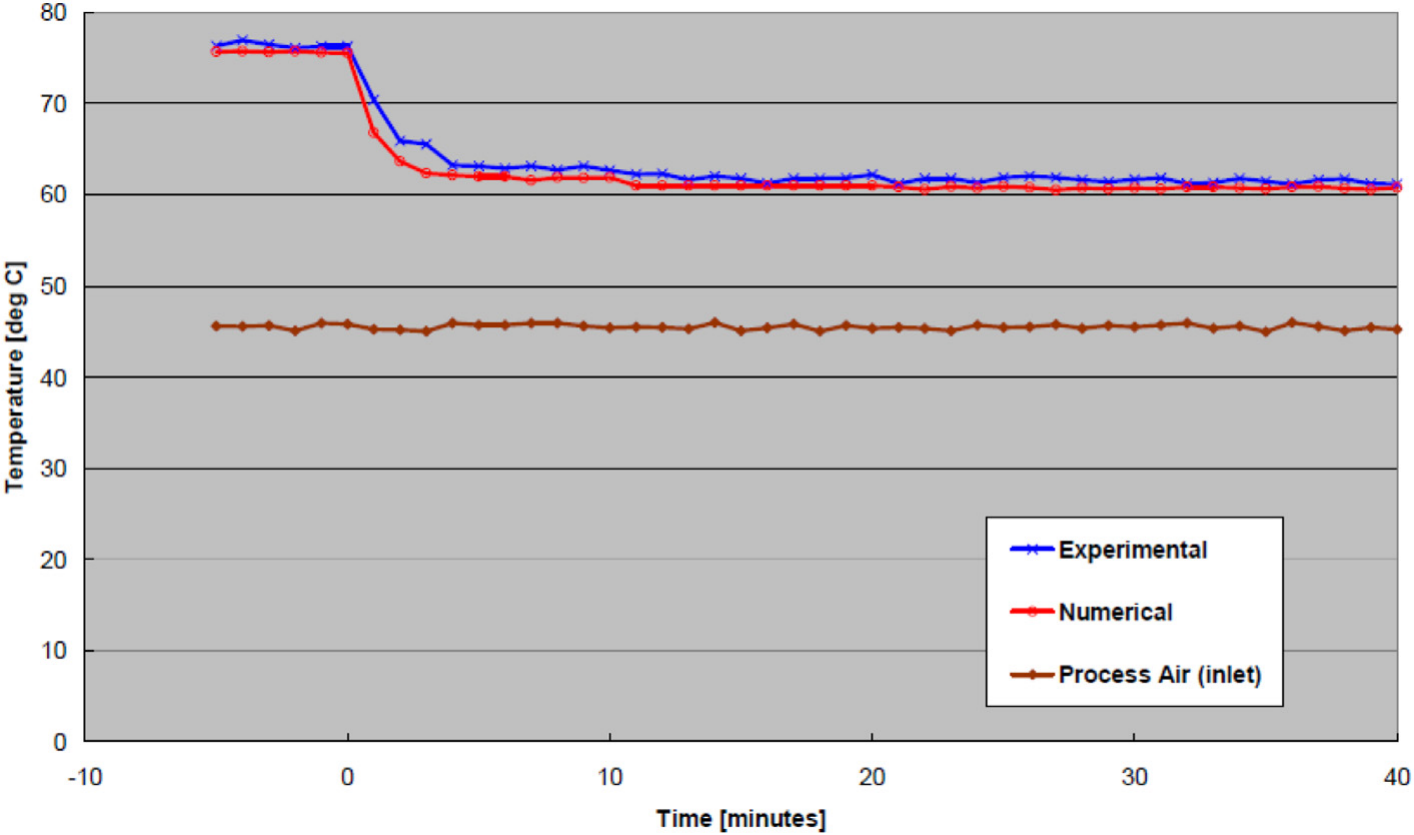
Outlet process air temperatures from increased process airflow rate.

**Figure 9:**
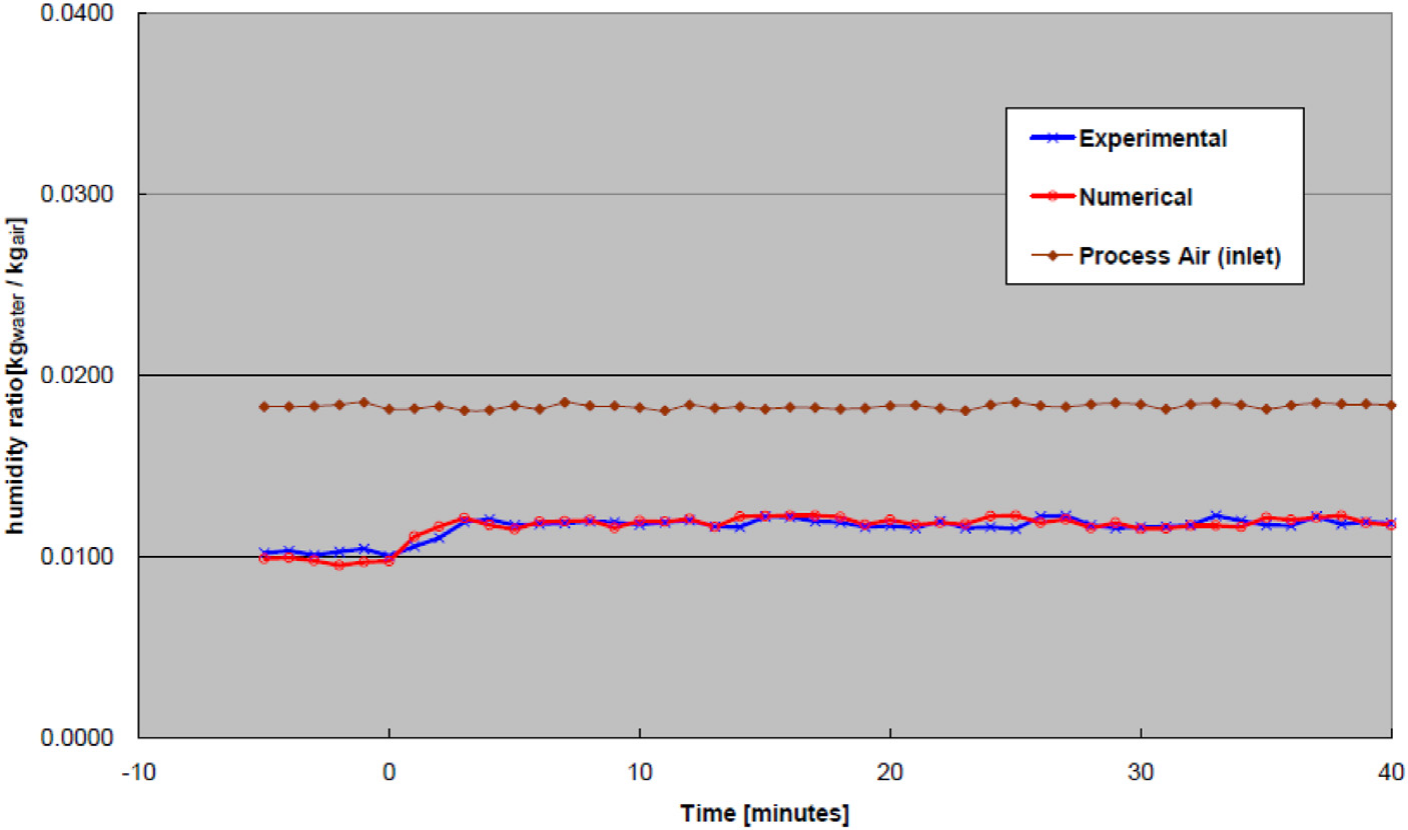
Outlet process air humidity ratios from increased process airflow rate.

Examples from the effect of wheel speed increase are shown in Figure 6 (outlet process air temperature) and Figure 7 (outlet process air humidity ratio). Similarly, examples from the effect of process air flow rate increase are shown in Figure 8 (outlet process air temperature) and Figure 9 (outlet process air humidity ratio). As seen from these Figures, the process air temperature and humidity ratio of numerical model curves generally look like those of the experimental results. In general, the transient effect due to changes of wheel speed tends to oscillate whereas the effects due to changes of airflow ate would fluctuate more sinuously. Nonetheless, the transient effects from all runs (increase to regeneration air temperature, decrease to regeneration air temperature, increase to wheel speed, decrease to wheel speed, increase to process air flow rate, and decrease to process air flow rate) were validated statistically by ways of RMSE, as shown in Table 2. Also tabulated in the Table is the required transient time to reach steady-state under each operating condition.

**Table 2:** Validation results under transient time for all runs and parameters.

Run	Air stream	Parameter	Average value	RMSE	Initial value	Steady-state value	Transient time
Increase to regenerate air temperature	Process air	Temperature	54.8 °C	5.95 °C	35.0 °C	59.8 °C	22 min
		Humidity ratio	.0127	.0013	.0183	.0122	20 min
	Regeneration air	Temperature	48.6 °C	6.57 °C	35.0 °C	41.3 °C	20 min
		Humidity ratio	.0263	.0030	.0183	.0350	5 min
Decrease to regenerate air temperature	Process air	Temperature	46.8 °C	1.08 °C	59.8 °C	32.7 °C	38 min
		Humidity ratio	.0124	.0001	.0122	.0175	38 min
	Regeneration air	Temperature	35.4 °C	1.13 °C	41.3 °C	26.8 °C	36 min
		Humidity ratio	.0230	.0010	.0350	.0100	26 min
Increase to wheel speed	Process air	Temperature	60.3 °C	0.68 °C	59.8 °C	60.7 °C	5 min
		Humidity ratio	.0120	.0004	.0126	.0118	4 min
	Regeneration air	Temperature	51.3 °C	6.25 °C	47.5 °C	26.8 °C	12 min
		Humidity ratio	.0343	.0010	.0340	.0350	10 min
Decrease to wheel speed	Process air	Temperature	58.7 °C	0.50 °C	59.8 °C	57.6 °C	10 min
		Humidity ratio	.0123	.0006	.0117	.0127	5 min
	Regeneration air	Temperature	51.3 °C	8.16 °C	47.5 °C	26.8 °C	10 min
		Humidity ratio	.0345	.0011	.0350	.0340	8 min
Increase to process air flow rate	Process air	Temperature	62.3 °C	1.83 °C	70.3 °C	61.3 °C	12 min
		Humidity ratio	.0105	.0002	.0096	.0110	10 min
	Regeneration air	Temperature	50.6 °C	7.04 °C	52.3 °C	49.8 °C	10 min
		Humidity ratio	.0340	.0004	.0340	.0350	10 min
Decrease to process air flow rate	Process air	Temperature	68.5 °C	1.36 °C	62.4 °C	74.9 °C	15 min
		Humidity ratio	.0108	.0005	.0118	.0095	13 min
	Regeneration air	Temperature	50.7 °C	8.67 °C	49.4 °C	52.3 °C	15 min
		Humidity ratio	.03521	.0012	.0357	.0344	15 min

It can be seen from the transient (Table 2 and Figures 4, 5, 6, 7, 8, and 9) and steady-state (Table 1) results that the numerical model represents the transient and steady-state response of the rotary desiccant wheel to a reasonably close approximation. As shown in Table 2, the transient time typically requires 10–15 min to reach steady-state when the wheel speed or process air flow rate is increased or decreased. The transient time is much longer (26–38 min) when the regeneration air temperature is decreased than the time (20–22 min) it takes to reach steady-state when the regeneration air temperature is increased.

## 7 Parametric analysis

From the validated finite-difference numerical model, a parametric analysis was performed to determine the optimal operating characteristics and factors that affect the transient response of the rotary wheel. The desiccant wheel was analyzed over different ambient temperature, ambient humidity, wheel speed, process air flow rate, and wheel-split with regeneration air temperature. A base-case air flow rate was set to 600 fpm (feet-per-minute) or 3.048 m/s (meters per second) entering both the process air side and regeneration air side of the rotary wheel. The velocity-based air flow rate is the conventional practice in HVAC (heating, ventilating, and air-conditioning) applications rather than mass-based air flow rate that is difficult to control. It was expected that the difference of regeneration air temperature would generate the most significance since the primary driving potential for moisture transfer depends on the amount of dry heat within the wheel. Recall Table 2 and Figures 4 and 5 had shown the longest transient response time for step-changes of the regeneration air temperature.

**Figure 10:**
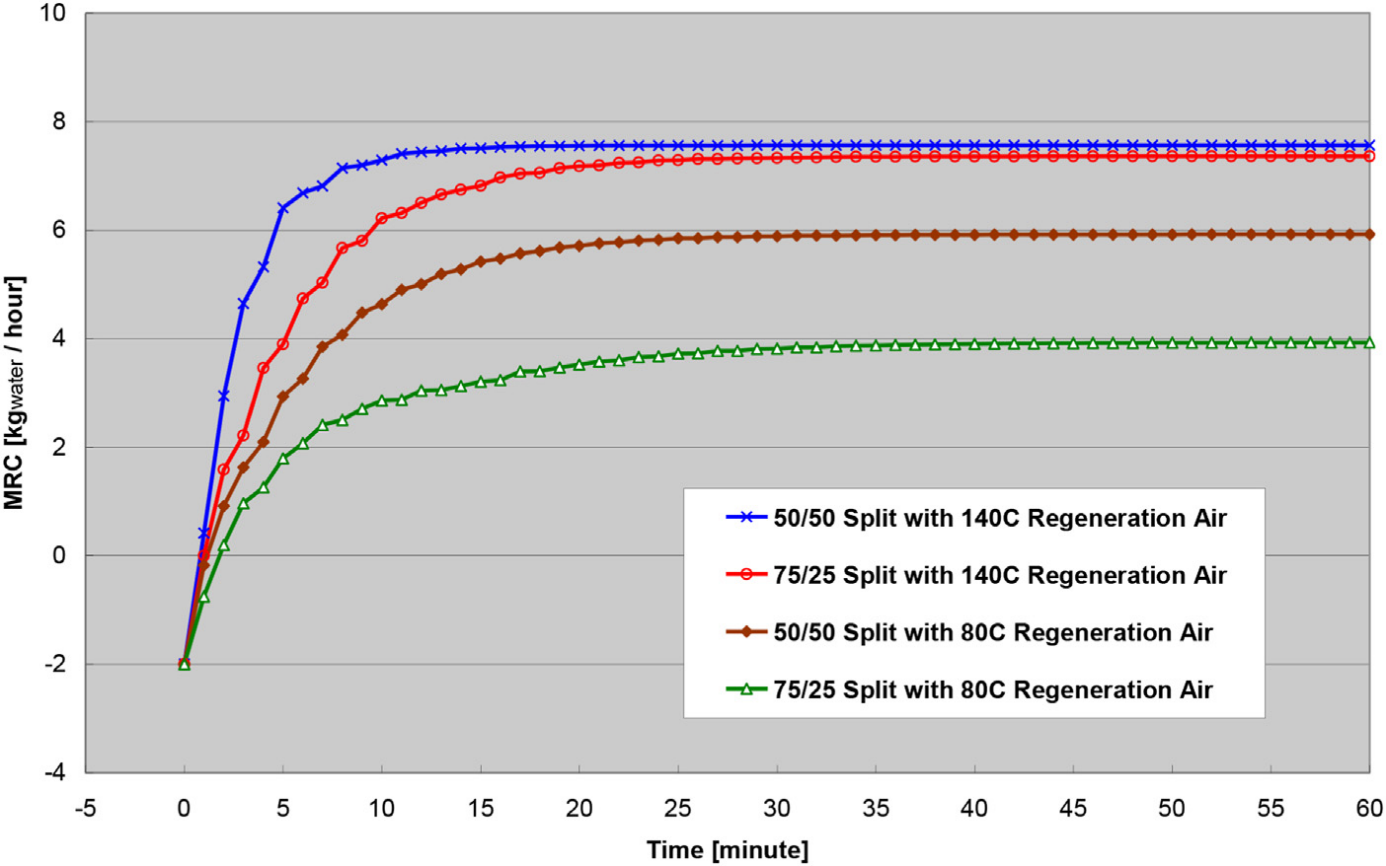
Transient response of different wheel-split and regeneration air temperature.

Figure 10 shows the transient response from the combination of different wheel-split and regeneration air temperature. It can be seen that the higher of the regeneration air temperature, the more moisture removal capacity (MRC) can be achieved at steady-state. At the same time, the transient response period is longer when the regeneration air surface area is reduced to 25% of the total wheel surface area (i.e. 75/25 wheel-split). It is faster to reach steady-state when the regeneration airstream and process airstream flow through a balanced 50/50 wheel-split.

Another key finding from the Figure is the difference of steady-state MRC between balanced and unbalanced wheel-splits at high (140 °C) or low (80 °C) regeneration air temperatures. The difference of steady-state MRC is larger at 80 °C regeneration airstream whereas the difference is smaller at 140 °C regeneration airstream. The implication is that a larger amount of thermal energy is lost at 50/50 wheel-split when the regeneration airstream is super-heated. An unbalanced 75/25 wheel-split may be sufficient enough to deliver the desired MRC at nearly half of the required thermal energy (i.e. only 25% of the wheel surface area being heated by the regeneration airstream vs. 50% of the area to be heated). Hence, a 75/25 wheel-split is more desirable for super high regeneration air temperature when extra thermal energy is demanded to deliver increased regeneration air temperature and MRC.

When the regeneration air temperature is lower (80 °C), a balanced 50/50 wheel-split not only can deliver much higher steady-state MRC (nearly 6 kg_w_/h vs. 4 kg_w_/h) but also shortens the transient response time (16 min vs. 24 min), as shown in Figure 10. When the regeneration airstream is heated by a renewable (i.e. rejected heat from the cooling cycle) energy source, the regeneration air temperature is lower. A balanced wheel-split is much more preferable to deliver sufficient thermal capacitance (kg_w_/h) while minimizing regeneration energy loss. In other words, the increased wheel surface area (from 25% to 50%) being heated by the regeneration airstream uses the maximum heat capacity of the regeneration airstream while minimizing heat loss to the atmosphere. More heat would be released to the atmosphere if the regeneration air temperature is much higher than the inlet process air temperature.

**Figure 11:**
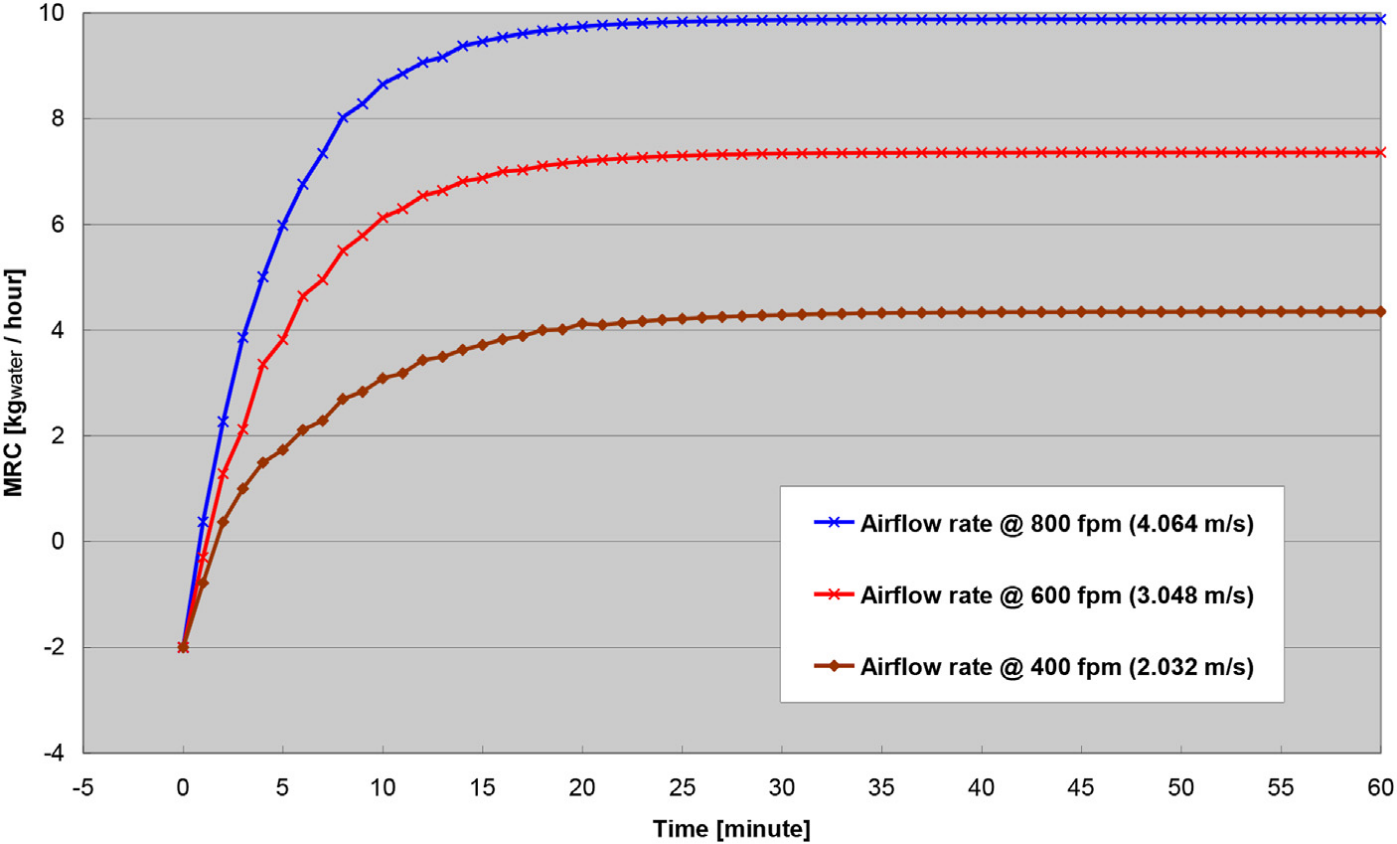
Transient response of different airflow rate.

The transient response at different air flow rate is shown in Figure 11. Both the process air flow rate and the regeneration air flow rate are increased or decrease concurrently from 400 fpm to 800 fpm. Illustrated in the Figure is the obvious result that the higher of the air flow rate, a higher of the steady-state MRC is achieved, as well as the shorter of the transient response time. Although it is advantageous to increase the air flow rate for higher MRC and shorter transient time, a practitioner should not be fooled to believe that the higher of the air flow rate would deliver the better result. There are two shortfalls to increased air flow rates that are not shown in the Figure. First, the fan power required to move air through the rotary wheel typically increases exponentially with increased airflow speed. Secondly and more importantly, Figure 11 was simulated under a constant regeneration air temperature. In real practice, the regeneration air temperature would decrease with increased air flow rate without adding additional heat energy. Recall in the previous result (Figure 10), decreased regeneration air temperature would lower the MRC and prolong the transient response. Hence, the penalties derived from increased air flow rate often far outweigh the benefit of increased MRC.

**Figure 12:**
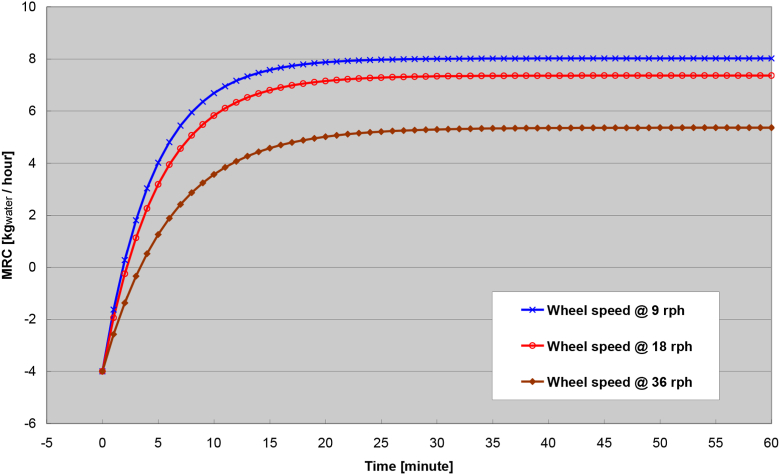
Transient response of different wheel speed.

The transient response at different wheel speed is shown in Figure 12. With a base-case scenario of the process and regeneration air flow rate at 600 fpm, the wheel speed is varied from 9 to 36 rph (revolutions per hour). The Figure showed that the slower of the wheel speed, the higher of the steady-state MRC is achieved. However, a caution should be noted that the optimal wheel speed depends on other operating conditions such as the regeneration air temperature, airflow rate, and ambient humidity to maximize the mass transfer of the rotary desiccant wheel. In plain words, higher wheel speeds would deliver more energy transfer and less mass transfer. Slower wheel speeds would deliver less energy transfer and more mass transfer. When the regeneration air temperature is higher, the wheel speed can be adjusted higher. When the airflow rate is higher, the wheel speed can also be adjusted higher.

**Figure 13:**
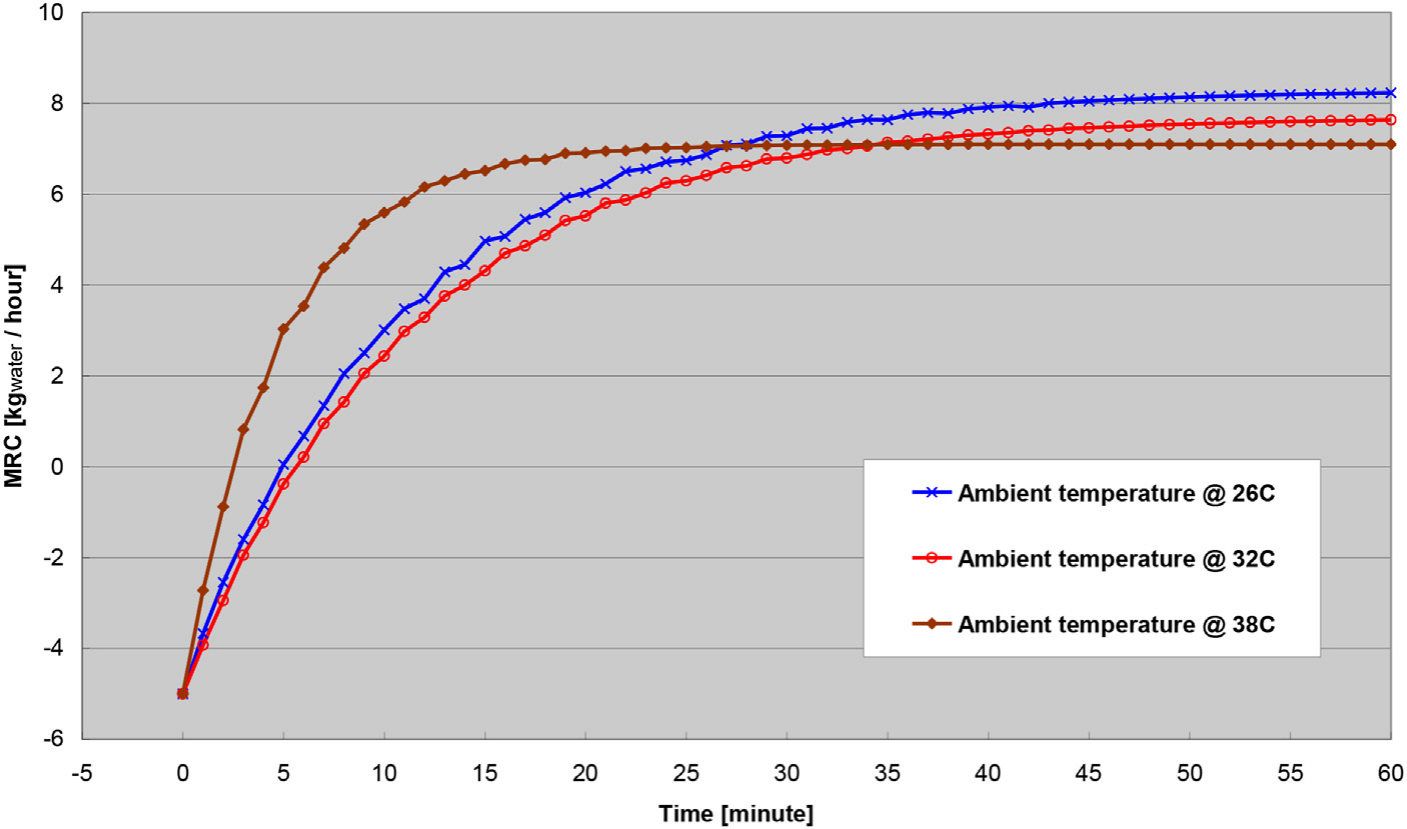
Transient response of different ambient temperature.

The transient response at different ambient temperature is shown in Figure 13. The Figure showed the lower of the ambient temperature, the higher of the steady-state MRC is achieved. However, more transient response time is needed to achieve the higher steady-state MRC with the lower ambient temperature. The reason for the prolonged transient response time with lower ambient temperature is due to the higher temperature differential between the process and regeneration airstream. It must also be noted that the result of this Figure was simulated at constant ambient humidity, regeneration air temperature, air flow rate, and wheel speed. In real life, ambient humidity usually rises with higher ambient temperature. It is presumed that more moisture can be removed (i.e. higher MRC) from higher ambient humidity ratio, where higher ambient humidity ratio is usually attached with higher ambient temperature which would have reduced the temperature differential between the process and regeneration airstream, and the subsequent MRC. The presumption from the factor of ambient humidity would be examined next.

**Figure 14:**
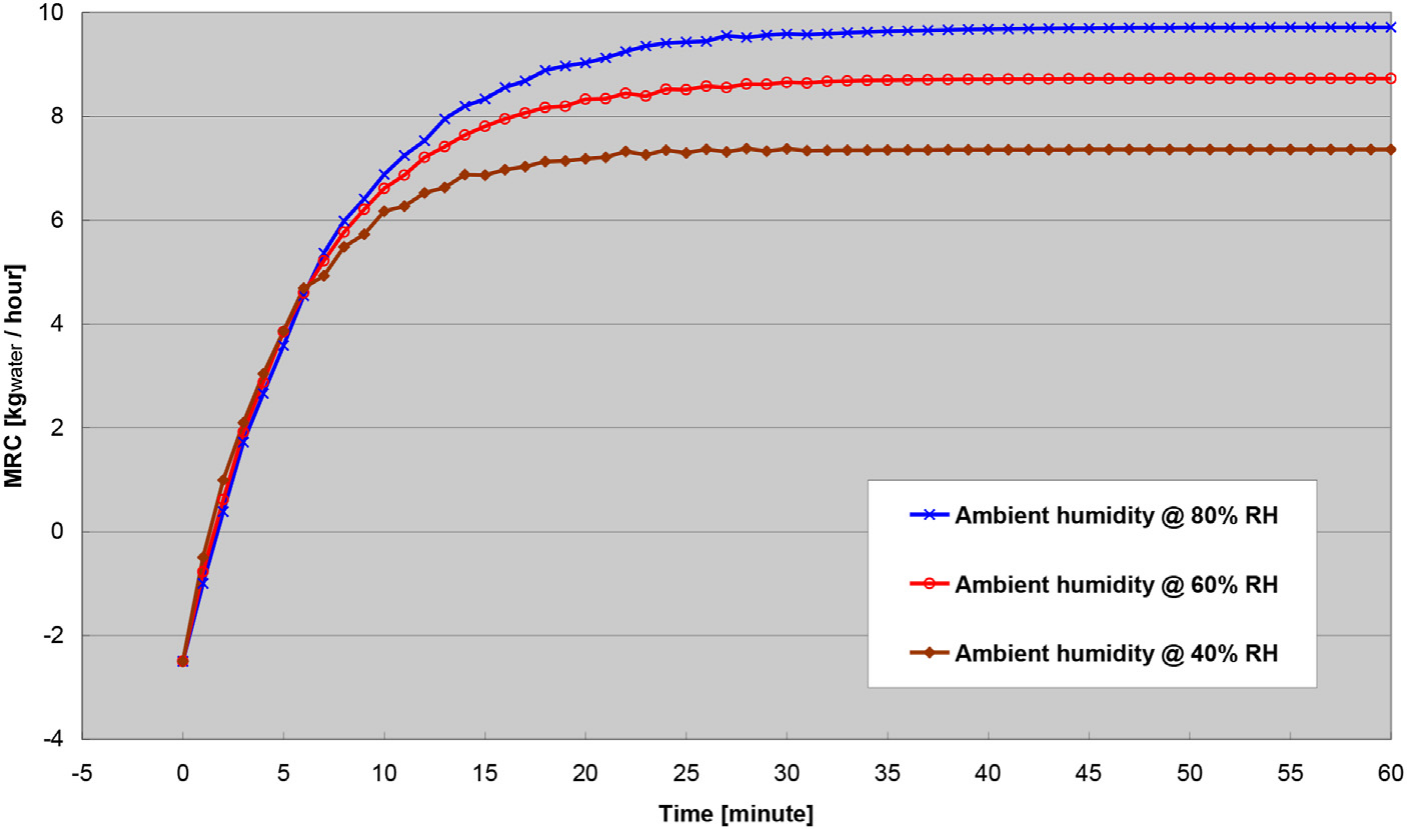
Transient response of different ambient humidity (relative humidity).

The ambient humidity is of concern much more than the effect from ambient temperature because ambient humidity can vary much more greatly over time. The transient response with respect to different levels of ambient humidity while keeping ambient temperature constant is shown in Figure 14. The Figure showed the higher of the ambient humidity, the higher of the steady-state MRC is achieved. As expected and presumed, the higher of the steady-state MRC from higher ambient humidity is simply caused by a greater magnitude of moisture content in the ambient (process) airstream to begin with. Similar to the previous result (ambient temperature), more transient response time is also needed to achieve the higher steady-state MRC. The longer transient period is due to the slow development of the moisture gradients within the surface particle that is caused by the solid-side mass resistance of the rotary wheel.

## 8 Conclusions and recommendations

The parametric analysis has demonstrated how ambient conditions (temperature and humidity), airflow rates, and operational characteristics (wheel-split and wheel speed) can greatly affect the performance (steady-state MRC and its transient response time) of the rotary desiccant wheel significantly. The greater of the temperature or humidity differential between the process and regeneration airstreams, the higher of the steady-state MRC may be achieved but associated with prolonged transient response time. From the perspective of the process air side, the rotary wheel works best on rainy days (i.e. high ambient humidity). However, it becomes tricky temperature-wise. Although a lower ambient temperature would increase the temperature differential between the process and regeneration airstreams if the regeneration temperature is at constant, energy loss from the rotary transfer leakage is also greater with the higher temperature differential. In addition, lower ambient temperatures usually come with lower ambient humidity on non-rainy days. From these two caveats, higher ambient temperature is preferred for optimal performance of the rotary wheel whereas the transient response time is also much shorter.

From the perspective of the regeneration side, although a higher regeneration air temperature would increase the temperature differential for higher steady-state MRC, the additional energy input required to increase the regeneration air temperature may not worth it. Lower regeneration air temperature can be compensated with increased wheel surface area (i.e. using 50/50 wheel-split). Hence, the optimal wheel operation shall adapt 50/50 wheel-split with whatever the regeneration air temperature that came from the rejected heat of the cooling system via heat exchangers. Standard airflow rate at 600 fpm and wheel speed at 18 rph is sufficient enough for typical operation unless the regeneration airstream temperature drops substantially. Since the regeneration air temperature is directly related to the output of the renewable energy (i.e. rejected heat), a decrease of the renewable output also means a decrease of the cooling demand where the MRC demand is lower. Hence, an increase of the airflow rate may still not be needed to satisfy the reduced MRC demand.

Using the recommended control strategy, the energy saving due to installed rotary desiccant wheel can be as high as 11.2% during hot and humid summers. The saving in winter is minimal because of low cooling demand and lower humidity of the winter ambient. However, the improved thermal comfort from dehumidified indoor space cannot be quantified. Because of frequent raining in northern Taiwan (where Taoyuan is situated), the feasibility of a hybrid-photovoltaic system as an energy source to heat regeneration airstream is minimal, taking into consideration of the prolonged payback time. The irony of using solar radiation as suitable energy source to heat regeneration airstream is that solar panels work best in desert-like climates (i.e. sunny and dry) where desiccant cooling may not be needed. Implication of the parametric analysis showed that, with proper control strategies, a rotary desiccant wheel using rejected heat as a renewable energy source to heat regeneration airstream would work very well in a hot and humid climate.

Shortcomings and limitations exist in most experimental researches. With no exception, there are areas where additional work may be performed for more improvements to the modeling of rotary desiccant wheels. First of all, the adapted estimation methodology did not take into account of the effect from auxiliary components (i.e. cooling coils, heat pipes, etc.) where future study may integrate these components with a more sophisticated model to the combined effects. Secondly, the experimental work was limited by one existing sorption material, the silica gel as its desiccant wheel. Future researches may test the effects of other sorption materials such as zeolites and molecular sieves in laboratory setups to determine their associated transient responses. Experimental trial of different wheel sizes (diameter and depth) combined with different sorption materials is also more feasible in laboratory setups.

## Declarations

### Author contribution statement

Hung-Yi Tsai: Conceived and designed the experiments; Performed the experiments; Contributed reagents, materials, analysis tools or data.

Chung-Tai Wu: Analyzed and interpreted the data; Wrote the paper.

### Funding statement

This research did not receive any specific grant from funding agencies in the public, commercial, or not-for-profit sectors.

### Data availability statement

The data that has been used is confidential.

### Declaration of interest’s statement

The authors declare no conflict of interest.

### Additional information

No additional information is available for this paper.

## Footnotes

1 Supplement data and codes are provided in a separate Data in Brief journal: *In situ* real-time dataset as compared to the predicted thermal wheel performance of an energy recovery system
